# Is Infant/Toddler Anemia a Problem across Rural China? A Mixed-Methods Analysis

**DOI:** 10.3390/ijerph15091825

**Published:** 2018-08-23

**Authors:** Lei Wang, Yonglei Sun, Buyao Liu, Lijuan Zheng, Mengjie Li, Yu Bai, Annie Osborn, Maggie Lee, Scott Rozelle

**Affiliations:** 1 International Business School, Shaanxi Normal University, Xi’an 710069, Shaanxi, China; wangleiml@163.com (L.W.); limengjie95@163.com (M.L.); 2 Center for Experimental Economics of Education, Shaanxi Normal University, Xi’an 710069, Shaanxi, China; sunyongleiceee@163.com (Y.S.); liubuyaoceee@163.com (B.L.); zhenglijuanceee@163.com (L.Z.); 3 Rural Education Action Project, Stanford University, Stanford 94305, CA, USA; anniepellosborn@gmail.com (A.O.); maggieleeeeeee@gmail.com (M.L.); rozelle@stanford.edu (S.R.)

**Keywords:** infants/toddlers, anemia, rural China, mixed-methods

## Abstract

In the past, iron-deficiency anemia in children has had a widespread presence in rural China. Given the recent economic growth in China, it is unclear if anemia among infants/toddlers remains a problem. The objective of this study is to measure the anemia rate in rural Chinese infants/toddlers across four major subpopulations and attempt to discover the sources of anemia. We use a mixed-methods approach combining quantitative data on 2909 rural Chinese infants/toddlers and their families with qualitative interviews with 84 caregivers of infants aged 6 to 30 months. Quantitative analysis indicates that the overall prevalence of anemia (43%) within sampled infants/toddlers was high, especially in comparison to the low rates of stunting (2–5%), being underweight (2%), and wasting (2–4%). These findings suggest that in rural China, anemia stems from the poor quality of the diets of infants/toddlers, rather than insufficient quantities of food being consumed. Qualitative analysis illustrates the factors that are contributing to anemia. Caregivers do not understand the causes of this condition, the symptoms that would lead one to recognize this condition, or the steps needed to treat their child with this condition. The findings offer a comprehensive understanding of the limited awareness of anemia among rural Chinese caregivers.

## 1. Introduction

Iron-deficiency anemia is a micronutrient deficiency common among infants in developing countries. The first few years of life, during which the brain is particularly vulnerable to biological, environmental, and psychosocial influences, are especially important for healthy childhood development [1,2,3]. Thus, anemia occurring in infants and toddlers can have long-term ramifications, most notably affecting cognitive and psychomotor developmental delays [1,4,5,6,7,8,9,10]. The delays resulting from anemia can affect numerous outcomes later in life, including educational attainment, employment, and earnings [3,11,12,13,14].

Several studies have found particularly high rates of anemia among infants in poor, remote rural areas of China, especially mountainous regions. In southern Shaanxi province, roughly half of infants aged 6–12 months living in villages located in nationally designated poverty counties were anemic (48.8% [15]; 50.5% [16]). In one autonomous Tibetan prefecture of the Qinghai province, 64.7% of children aged 6–35 months living in the prefecture’s nationally designated poverty counties suffered from anemia [17]. In poor rural areas of Guangxi province, 22.6% of children aged 0–24 months were anemic [18]. To contextualize these numbers, iron-deficiency anemia rates for toddlers aged 1–3 years in the U.S. range from 0.9% to 4.4% [19]. In high-income countries worldwide, there is an 11% prevalence rate for anemia among children under 5 years old [20]. Hence, the proportion of anemic infants and toddlers in poor, remote areas of rural China is distinctly high by comparison.

However, China’s rural infants and toddlers do not solely inhabit poor, remote areas of the country. According to the Chinese household registration system, anyone who possesses a rural *hukou* (residency status assigned at birth) is a rural citizen, regardless of where they actually live [21]. In practice, this means that in addition to living in poor, remote, mountainous regions, rural Chinese populations can also be found in villages in the plains (where resources are generally less scarce than in the mountains), in resettlement communities (residential communities created by the state to consolidate the scattered poor rural populations living in mountainous villages by providing these households with subsidized housing [22]), and in migrant communities located in large urban centers [23]. In fact, only about 26% of China’s rural infants and toddlers actually live in poor mountainous regions [24]. In contrast, 29% of rural infants and toddlers are growing up in plains villages and 13% are growing up in migrant communities. Including the rural infants and toddlers in resettlement villages (1.4%), more than two-thirds (69%) of China’s rural infants and toddlers and nearly half of all infants and toddlers (49% = 69% of 71% of China’s total number of children who have permanent rural residency [24]) live in one of these four subpopulations (western China rural communities, resettlement communities, central China rural communities, or migrant communities).

Given how much the different environments rural infants and toddlers inhabit can vary across space, it is reasonable to investigate whether anemia rates among infants and toddlers also vary across different rural communities. If infant anemia rates are different across these communities, it is important to identify what the caregivers of the infants and toddlers in these other subpopulations with lower anemia rates are doing differently, or whether the discrepancies are due to differences in the availability of resources. On the other hand, if anemia rates are similar despite the differences in rural community structure and location, it is important to determine whether the sources of anemia are different in distinct communities. For instance, does the understanding of anemia, and of its sources, symptoms, and treatments, vary across these populations?

The current literature on anemia rates in rural China does not allow us to answer these questions. Previous research has tended to focus on research venues with relatively narrow geographic ranges (e.g., just one prefecture [17] or a small number of counties in one part of one province [15]). Those studies that look at a wider geographic range (an entire province, for instance) still usually only target one kind of rural community, namely, rural Chinese living in poor, remote, mountainous areas [18,25]. To our knowledge, only one study has looked at infant anemia rates in urban migrant communities [26]. The prevalence of anemia in this study was 36.6% for migrant infants aged 6 to 23 months. The sample in this study was from a small coastal city (Pinghu city, Zhejing province). Consequently, the findings were not representative for migrant communities in big cities in China—the types of cities that have large concentrations of rural families who have migrated to find jobs [27]. We know of no research in recent years that has used standardized protocols to compare infant anemia rates across four different kinds of rural communities.

The current study seeks to fill some of the gaps in the existing literature on infant anemia in China. The overall goal is to understand, for different kinds of rural communities in China, the severity of infant anemia and to identify caregiver behaviors that contribute to (or help ameliorate) the problem. We achieve this goal by tracking anemia rates of infants/toddlers across different kinds of rural communities and in different geographic regions of China, allowing for a direct comparison of infant anemia rates in rural China. After establishing prevalence rates across China, we use the remaining quantitative part of the paper and qualitative interviews to seek to identify the sources of anemia. To do so, we look at other indicators of health and nutrition, including stunting, underweightness, and wasting, as proxies for understanding whether anemia is a fundamental problem of overall food insecurity/poverty or if it is appears to be mainly an issue of micronutrient deficiencies. Finally, in the qualitative part of the paper, we aim to identify which of the several possible sources of anemia are responsible for the nutrition deficiencies in rural China. Throughout the paper, we observe whether there are any systematic differences across the different rural subpopulations.

The rest of this paper is organized as follows. The next section describes our sampling methods, qualitative survey, and both quantitative and qualitative analysis strategies. The third section presents the results of the quantitative analysis. The fourth section reports on our qualitative investigation. The fifth section offers discussion and conclusion.

## 2. Methods

### 2.1. Study Location and Sample Selection

This study uses two types of data sets collected during six different survey efforts. Five different data sets collected in five different provinces in China are used in the quantitative analysis of this study. An additional data set of interviews is used for the qualitative analysis.

#### 2.1.1. Quantitative Data Sets

Given the goal of this study is to try to best understand infant and toddler nutrition across major subpopulations in rural China, the five quantitative data sets focus on one or more of four rural subpopulations: western China rural communities (hereafter referred to as “western rural communities”), resettlement communities, central China rural communities (hereafter referred to as “central rural communities”), and/or migrant communities. To classify our sample sites into one of these four categories, we make several assumptions about the distribution of these rural populations throughout China. We assume that western rural communities are primarily located in the twelve westernmost provinces of China, namely Xinjiang, Tibet, Qinghai, Ningxia, Yunnan, Sichuan, Guangxi, Guizhou, Gansu, Shaanxi, Inner Mongolia, and Chongqing. We also assume that central rural communities are chiefly located in eight central provinces, specifically Henan, Hubei, Hunan, Anhui, Jiangxi, Shanxi, Jilin, and Heilongjiang. Resettlement communities, by policy, are located in the same provinces as western rural communities. For the purposes of our study, we define migrant communities as groups of rural families living in China’s first-, second-, and third-tier cities.

While it is difficult to precisely divide China by regions (or provinces or provincial groups) into these subpopulations, since there are mountainous rural villages in central China and (likewise) since there are plains rural villages in western China, we still use this breakdown to produce a rough approximation of the share of rural Chinese infants and toddlers that are in each of the rural communities. Given the assumptions of the matches between community types and geographical regions, we estimate the number (and share) of infants and toddlers in each rural community type out of a total number of rural infants and toddlers aged 0 to 3 years (45 million in 2015 [27]). (According to the 2015 Micro-Census, China’s population was 1,374,619,936 and the share of 0–3-year-olds was 4.6% in 2015. We used the 2010 Census [28] to calculate the share of the population with rural hukou (70.86% of the total, which equals 974,087,753). We then used data from the 2015 Micro-Census to estimate the number of 0–3-year-olds with rural hukou (4.63% of the total, which equals 45,055,597).) In simplest terms, we estimate that there are 12 million infants and toddlers in western rural communities and that they account for 26% of all rural infants and toddlers in China. This number is estimated as the number of infants and toddlers in 12 western China rural provinces, minus the number of infants and toddlers in western resettlement communities (see below for the number in these communities) and minus 30% of the infants and toddlers who moved with their parents to migrant communities. According to our estimates, there are also 13 million infants and toddlers in central rural communities (29% of all rural infants/toddlers), which is estimated as the total number of rural infants and toddlers in central China’s eight provinces minus 37% of the infants and toddlers who moved with their parents to migrant communities.

Likewise, if one out of 36 rural migrant families took their child to the city, it would suggest that approximately 6 million infants and toddlers are in migrant communities. We computed these numbers by first obtaining the total number of China’s migrant population, which is defined as individuals who have moved out of their original province/county of residency and into urban areas. According to the 2015 Micro-Census, there were 246,656,258 migrants in that year. We then used the 2016 China Migrant Population Development Report [29] to calculate the number of migrants with rural hukou (84.40% of the total, which equals 208,177,882 individuals). Finally, we estimated the number of 0–3-year-olds migrants with rural hukou (2.81% of total rural migrants, which equals 5,839,663 individuals; this means one out of 36 rural migrant families took their children to cities). Accordingly, this would account for 13% (6 million divided by 45 million) of all rural infants and toddlers in China.

Finally, we estimate that there are 600,000 infants and toddlers in resettlement communities. This number is calculated by using the rough estimate that during the 11th, 12th, and 13th Five Year Plans for poverty alleviation and relocation, 12 million individuals moved into resettlement communities [30,31,32]. If 5% of this total figure is individuals aged 0 to 3 years, this would mean that there are 600,000 infants and toddlers living in resettlement communities.

A summary of the distribution and locations of sample households in each of these data sets can be found in Table 1. In total, 2909 infants aged 6 to 30 months are sampled in the data sets used for quantitative analysis. The breakdown of this sample shows that while most of the sample points are in western rural communities (84% of our sample), there are still 16% in the other three types of communities. In the paper, we avoid overweighting western rural communities (and underweighting resettlement communities, central rural communities, and migrant communities) by using weights that are created from the population shares. We calculate sampling weights for each observation using the following method. The proportions for each subpopulation in rural China are 37.7% for western China rural communities, 1.4% for resettlement migration communities, 42.0% for central China rural communities, and 18.8% for migrant communities. We calculate the sampling weights using the following formula: sampling weight = proportion of subpopulation in total population/proportion of subpopulation in sample. In our data, the subpopulation proportions are the following: 84.0% for western China rural communities, 4.6% for resettlement migration communities, 4.4% for central China rural communities, and 7.0% for migrant communities. The sampling weight each subpopulation is as follows: 0.44 for western China rural communities (which is equivalent to 37.7%/84%), 0.3 for resettlement migration villages (which is equivalent to 1.4%/4.6%), 9.55 for central China rural communities (which is equivalent to 42%/4.4%), and 2.96 for migrant communities (which is equivalent to 18.8%/7%).

The first two quantitative data sets describe populations living in western rural communities. Data were collected from villages in randomly selected nationally designated poverty counties in Shaanxi province (in data set 1) and Hebei and Yunnan provinces (in data set 2). (Nationally designated poverty counties, typically rural and mountainous, are categorized by the central government based on average per-capita income for the purposes of targeting poverty-alleviation programs [33].) Shaanxi province, located in northwestern China, is ranked 13 out of 31 in terms of provincial GDP (Gross Domestic Product) per capita (from high to low). Hebei province, located in eastern China, is ranked 19th. Yunnan, the southwesternmost province in China, is ranked 30th [34].

Sample households of each province were selected in the same manner across the data sets. After randomly selecting a nationally designated poverty county in a given province, we listed all townships in that county (excluding those townships where the county seat was located). A random village was then selected from each listed township. Finally, we obtained birth records from sample village family planning officials, and sampled all households with children between the ages of 6 to 24 months (in Shaanxi) or between the ages of 6 to 30 months (in Hebei and Yunnan). The first two data sets were collected between 2015–2016 and included 2442 observations.

The third quantitative data set describes populations living in resettlement communities. Data were collected from Henan province and the southern region of Shaanxi province. To select our sample households for this dataset, we used records from local family planning bureaus to obtain a list of all resettlement communities in Henan and southern Shaanxi where more than 50 babies aged 6 to 30 months lived. We choose two communities in Henan and two communities in Shaanxi. Within each community, 40 households with appropriately aged babies were randomly selected. In total, the third data set includes 135 observations.

The fourth quantitative data set describes populations living in central rural communities. Data collection took place in Henan province and the central region of Shaanxi province. The data collection process was similar to that used when sampling resettlement communities. After selecting one sample county that included central China rural regions both in Henan and Shaanxi, we randomly chose one sample village from a list of all villages in each sample county. Then, we randomly selected households with infants between the ages of 6–30 months from the family planning records of each village. In total, the fourth data set includes 128 observations.

The fifth quantitative data set describes migrant communities living in urban areas. These data were collected in Beijing, the national capital; in Zhengzhou, the provincial capital of Henan; and in Xi’an, the provincial capital of Shaanxi. Migrant communities in these urban areas were identified through records kept by the metropolitan family planning offices in each city. These same records allowed us to identify which communities had large numbers of households with children aged 6 to 30 months. Households in each of these communities were then randomly selected. In total, the fifth data set includes 204 observations.

#### 2.1.2. Qualitative Data Sets

The qualitative data set consists of a sample of twenty infants from each of the four rural subpopulations, for a total of 84 infants aged 6 to 30 months. Our qualitative data sampling frame was constructed using our quantitative samples from western rural communities, resettlement communities, central rural communities, and migrant communities in Shaanxi province. From each of our quantitative samples in each of these communities, we selected ten households with a child who was anemic and ten households with a child who was not. Due to concern that there would be households that would not comply with our requests during the survey (which in the end was not a problem), two extra households were sampled from both the central rural communities and the migrant communities, for a total of 84 households.

### 2.2. Data Collection

This section describes the methods used for the collection of both quantitative and qualitative data. Quantitative data include infant and toddler nutrition, health outcomes, and household characteristics. Data sets one and two were collected in 2016; all other data were collected in 2017. Qualitative data, collected in late 2017, include caregiver responses to questions about anemia, diet, and feeding behavior.

We collected detailed information on infant/toddlers, caregivers, and households. A survey was administered on infant/toddler, parent, caregiver, and household characteristics, including each infant/toddler’s age in months, gender, whether premature, maternal age, education, who was the primary caregiver, and household economic status measured by the level of household’s assets (household asset index) and whether the household receives social security support. Household asset index is constructed using polychoric principal components on the following variables: tap water, toilet, water heater, washing machine, computer, internet, refrigerator, air conditioner, motor or electronic bicycle, and car. We calculate overall summary statistics using sampling weights for each observation.

For every infant in our sample, we recorded anemia status. Hemoglobin concentrations were measured by nurses from Xi’an Jiaotong Medical School using the HemoCue Hb 201+ finger prick system (Hemocue, Inc., Ängelholm, Sweden). A child with a hemoglobin concentration below 110 g/mL was classified as anemic, as per the threshold recommended by the World Health Organization (WHO) [35].

We also collected anthropometric data for each child in order to determine whether or not children showed signs of stunting, underweightness, and wasting. We used the WHO growth charts [36] to construct three indicators: height-for-age *z*-scores (HAZ), weight-for-age *z*-scores (WAZ), and weight-for-height *z*-scores (WHZ). WAZ and WHZ are linked to recent or brief changes in diet, whereas HAZ is linked to the effects of longer-term, cumulative nutritional investments as well as illnesses [37]. Low HAZ scores indicate stunting, low WAZ scores indicate underweightness, and low WHZ scores indicate wasting (for all indicators, the cutoff for “low” is below—2 standard deviations under the mean [38]).

While infants were tested for anemia and had their anthropometrics measured, we also administered a survey to caregivers asking for basic household information. This included parental age (older or younger than 25 years), parental education levels (above or below 12 years), who the primary caregiver for the infant was (mother or other, typically the grandmother), whether the household received social security support, and the nature of each household’s assets.

Qualitative interviews were conducted by trained researchers from Shaanxi Normal University and Stanford University. Researchers interviewed the primary caregiver, usually the mother or the grandmother, of each household, where possible. When this was not possible, researchers interviewed the secondary caregiver, typically either the father or a grandparent. All interview subjects were asked if the interview could be recorded, and all but five gave permission. Regardless of whether the interview was recorded, detailed handwritten notes were taken during the interview.

Our qualitative interview comprised seventeen open-ended questions that can be divided into sets of questions to test several hypotheses. The first category of questions asked whether the caregiver knew what anemia, in general, was. The second category asked the caregiver to provide more in-depth knowledge about specific aspects of anemia: Did the caregiver know what caused anemia?; Did the caregiver know what effects anemia could have on their child? Did the caregiver know what the symptoms of anemia were—both outward physical symptoms or behavioral symptoms?; and, Did the caregiver know how to treat anemia? Finally, the third category of question probed at trying to identify other causes of anemia—e.g., the nature of the rural environment or the poor quality of the rural healthcare system—that might be leading to nutrition problems for the child, even if the caregiver had a complete understanding of anemia.

## 3. Results

### 3.1. Quantitative Results

In this section, we report on the results of the quantitative findings of the study. To do so, we first present descriptive statistics describing the average characteristics of the infants/toddlers and the families. Second, we report the results of the child health assessments, which include the analysis of the rates of anemia and the prevalence of stunting, being underweight, and wasting in the overall samples as well as in the four subpopulation samples. Third, we explore the health and nutrition status of the sample children as they age (that is, whether the rates of anemia rise or fall between the ages of 6–18 and 18–30 months). Finally, we examine the relationship between anemia status and the characteristics of the infants/toddlers and households in each of the study’s four rural subpopulations.

Descriptive statistics from the survey for each of the communities are shown in Table 2. We can see that the percentage of mothers who have attained at least 12 years of education in migrant communities (61%) is significantly higher than in other rural communities (western rural communities: 23%; resettlement communities: 36%; central rural communities: 32%). The rates differ significantly. We also find that a significantly greater percentage of primary caregivers in migrant communities and western rural communities are mothers (73%), compared to other rural communities (resettlement communities: 54%; central rural communities: 56%). The share of mothers in resettlement communities and central rural communities are statistically lower comparing to western rural communities (*p*-value < 0.01). The family asset index is also significant lower (−0.19) in western rural communities than in other rural communities (0.37, 0.48, and 1.23 for resettlement communities, central rural communities, and migrant communities, respectively; *p*-value < 0.001). The share of households who received social security support is almost the same across the four different rural communities.

Anemia rates for the sample individuals in all the rural communities included in this study are shown in Table 3. In total, 43% of all infants were anemic. Within the subpopulations, however, the point estimates of the share of infants that were anemic varied slightly, although the rates in all subpopulations were relatively high. The rates for the different communities ranged from 40% in western rural communities, 52% in resettlement communities, and 46% in central rural communities to 43% in migrant communities. Importantly, while the point estimates differed somewhat, the rates of anemia in the four subpopulations did not differ statistically.

The data also show that the rates of anemia fall as a child ages (between the time of infancy, ages 6 to 18 months; and toddlerhood, ages 18 to 30 months). The rate of anemia in infants for the overall sample was 43%. When we examine the anemia status of infants aged 6 to 18 months, we find that over half of the infants (51%) were anemic. As the infants get older, anemia prevalence drops: the anemia rate was 30% among infants ages 18 to 30 months (Table 4). However, it is important to note that the anemia rates were still high compared to those of healthy populations. Clearly, the rates of anemia were high for all subpopulations for all age ranges investigated in China.

While rates of anemia were high, stunting, underweightness, and wasting were uncommon in all communities (Table 3). Stunting prevalence was 5% in western rural communities, 5% in resettlement communities, 3% in central rural communities, and 2% in migrant communities. These differences were statistically insignificant. Underweight prevalence was 2% in western rural communities, 2% in resettlement communities, 2% in central rural communities, and effectively not present in migrant communities. Wasting prevalence was 3% in western rural communities, 2% in resettlement communities, 4% in central rural communities, and 4% in migrant communities. The differences for being underweight and wasting also were statistically insignificant.

So, who is anemic? When we run correlational analysis between anemia (yes = 1; no = 0) and the basic control variables in our sample (individual and family characteristics), we find three prominent results (Table 5). First, the age of an infant is negatively correlated with anemia. In other words, the older the infant is, the less likely it is that he or she is anemic (*p*-value < 0.001). Second, we do not find that household economic status is associated with anemia. In our analysis, we measure the household economic status by the level of a household’s assets (a measure of wealth) and whether the household receives social security support (a measure of poverty). The correlational analysis (Table 5) shows that there is no significant relationship between anemia and either variable (*p*-values = 0.557 and 0.752, respectively). Third, the location in which the child is living (as seen above in the descriptive statistics) is not correlated with whether or not the child is anemic (*p*-values = 0.754, 0.182, and 0.161 for western rural communities, resettlement migration communities, and central rural communities, respectively, comparing to migrant communities). Wherever the infants/toddlers live, even in urban areas, the rates of anemia are high.

### 3.2. Qualitative Results

Given the high levels of anemia across different subpopulations of rural communities, the qualitative section of this paper focuses on trying to identify the source of the problem. Why is it that in communities where average income is not that low and where macronutrients appear to be sufficient (that is, there is little stunting, underweightness, and wasting), anemia is so widespread? To answer this question, 84 participants from the quantitative study were randomly selected to participate in the qualitative study.

The qualitative analysis is organized around three hypotheses: Hypothesis 1: High rates of anemia are prevalent because caregivers do not know what anemia is. Hypothesis 2: High rates of anemia occur, not because people do not know what anemia is, but because they have a poor understanding of (or are misinformed about) how to address the problem. In fact, the second hypothesis can be broken down into four sub-hypotheses: (a) caregivers misunderstand the real cause of anemia; (b) caregivers misunderstand how anemia affects their child; (c) caregivers do not know how to assess whether their infants/toddlers have anemia (or caregivers do not know what the symptoms of anemia are); and/or (d) caregivers do not understand how to treat anemia. Hypothesis 3: Anemia is a problem because of constraints such as poverty or the absence of a functioning health care system, and not because caregivers do not understand what it is or how to address it. It is these limitations that keep caregivers from seeking a cure for their anemic child.

In this section, we will draw on the study’s qualitative interviews to shed light on this study’s main hypotheses about why anemia rates are so high in a country like China. Where appropriate, we compare differences in qualitative responses across rural communities to demonstrate situations when the factors behind high rates of anemia appear to be the same in all rural communities or when the sources of anemia appear to vary by subpopulation.

**Hypothesis** **1.*** High rates of anemia are prevalent because caregivers do not know what anemia is.*

“Have you heard of anemia?” When answering this basic question with an either yes or no response, nearly all interviewees (78 out of 84) across all subpopulations claimed they had at least heard of this condition. All interviewees (20 out of 20) in the western rural communities said “yes.” In no case did less than 87%of individuals in any given subpopulation (the lowest share was in the migrant subpopulation) say that they had heard of anemia. Therefore, in the purest sense, hypothesis 1 appears to be false: high rates of anemia are not due to the fact that caregivers do not know that anemia is.

**Hypothesis** **2.** *High rates of anemia occur, not because people do not know what anemia is, but because they have a poor understanding of how to address the problem.*

To examine hypothesis 2, we will treat each sub-hypothesis individually. The objective of the analysis in this section is to not only identify if poor information is a source of the anemia problem, but, more importantly, to discover exactly what the nature of the information problem is. There are four subsections in this part of the analysis.

****Sub-Hypothesis 2a.*** The misinformation is due to the fact that caregivers do not know what the real cause of anemia is.*

When asked what anemia is, 8 out of 84 interviewees correctly described that anemia was related to iron or hemoglobin deficiency.

*“(Anemia happens when) nutrition is poor, and there is an iron deficiency.”*

*(Mother, central China rural village, 1231128)*

*“Anemia is low levels of hemoglobin.”*

*(Grandmother, migrant community, 1221161)*

However, although a large share of the interviewees (across all subpopulations) had heard of anemia, it was evident that they did not know the real cause of the condition. Specifically, 22 caregivers said that although they had heard of anemia, they admitted that did not know what caused it. The rest of the caregivers (that stated that they had heard of anemia) offered incorrect, incomplete, or vague descriptions of the cause of anemia.

In one of the most commonly encountered responses, some (24 out of 84) caregivers knew that anemia was related to nutrition, but they did not have a concrete understanding of the relationship between the two:

*“I don’t really know (what anemia is). It should be something to do with lacking nutrition.”*

*(Grandmother, western China rural village, 1402116)*

One of the most frequently expressed pieces of knowledge (52 caregiver interviewees) was that anemia meant the child did not have enough blood. This may be due to the informal term for anemia in Mandarin: *pinxue*, which directly translates to “deficient blood.” In total, 10 interviewees *explicitly* mentioned insufficient blood when defining anemia.

*“The child’s nutrition isn’t good, which leads to the child not having enough blood.”*

*(Grandmother, western China rural village, 1402110)*

*“Anemia means there isn’t enough blood, which happens because of an iron deficiency.”*

*(Mother, resettlement community, 1112114)*

Three caregivers, all living in migration communities, said that anemia might be hereditary.

*“I can’t control (anemia in my child) by myself. It has to do (among other things) … with genes getting passed down, so who really knows?”*

*(Grandmother, migrant community, 1221304)*

About one-third of the interviewees (26 out of 84) admitted that they did not know what caused anemia. A slight plurality (9 out of 26) of those came from western rural communities. When asked for further insight into her understanding of anemia, one caregiver who had said that she knew anemia was not good for the child, responded vaguely:

*“I really don’t know. There’s way too much going on with kids, who knows?”*

*(Grandmother, western China rural village, 1406115)*

**Sub-Hypothesis** **2b.** *The misinformation is about caregivers not understanding how anemia affects their child.*

In our interviews with caregivers, it became clear that there were also misunderstandings about how anemia affects a child. Of the 84 interviews, 72 caregivers demonstrated their beliefs and understanding of the effects of anemia on their children. (Twelve caregivers said they “did not know” in response to relevant questions.) Of these 72 statements, responses in 50 of the interviews demonstrated fundamental misunderstanding. These responses were also fairly evenly spread across the households of the four rural subpopulations.

The statements about the impacts of anemia on infants can be divided into two general categories. In one category, caregivers simply did not understand (≈*n* = 16) the ramifications their children could face because of this condition. In the other, the misunderstanding of anemia’s effects appeared to derive from a misunderstanding of the source of the illness (≈*n* = 44).

In numerous cases, interviewees had a basic misunderstanding of the impacts of anemia on their children:

*“I don’t know—actually, it’s this, sometimes you squat down, and then after you get up you’re dizzy. That’s an example of the effect of anemia.”*

*(Father, western China rural village, 1221123)*

*“You don’t want to be anemic … there’s no energy. It’s like a car without oil.”*

*(Grandmother, migrant community, 1221304)*

In other cases, caregivers seemed to build on their misunderstanding of the root cause of anemia. Correct and incorrect information was mixed together in some responses:

*“(When a child is anemic), there’s dizziness, there’s not enough blood, (the child) won’t eat enough food, and (the child) won’t sleep well at night.”*

*(Mother, migrant community, 1221130)*

*“(Anemia happens when) there’s a lack of iron … and it’s easier to get sick and intelligence decreases.”*

*(Mother, resettlement community, 1112104)*

Only four caregivers thought that anemia was unimportant and that it had no effect on their children.

*“There’s no effect. Eating, sleeping, playing, whatever—they’re all fine.”*

*(Grandmother, central China rural village, 1231135)*

*“Generally speaking, my child catches cold frequently, gets sick easily. Their constitution is not so good. (Because of this), anemia shouldn’t have too much of an effect.”*

*(Mother, western China rural village, 1402130)*

**Sub-Hypothesis 2c.** *The misinformation is due to the fact that caregivers do not know how to assess whether their infants/toddlers have anemia (or the symptoms of anemia).*

Fatigue, reduced physiological endurance, and decreased cognitive performance are the main symptoms of anemia [39]. Other symptoms, specifically related to behavior, include a child feeling more lethargic than normal or being unable to focus. It is important to note, however, that in most cases (where anemia is not severe—which is the case in most of the individuals in this sample), the actual symptoms are not clearly visible or easy to detect.

Despite the difficulty of detecting anemia, more than half (48 out of 84) of the interviewees believed that anemia could influence a child’s development as seen through outward physical symptoms. Of these 48 interviewees, 35 caregivers specified that anemia could influence one or more of the following: height, weight, or constitution:

*“When you’re looking after the child, you have to pay attention to what’s going on. Sometimes the color of the child’s face isn’t quite right. (If) it’s a little yellow, that could be anemia.”*

*(Grandmother, western China rural village, 1406112)*

*“Their face will be yellow, and they’ll be short for their age.”*

*(Mother, resettlement community, 1112114)*

*“I saw on TV that anemic children are thin.”*

*(Father, resettlement community, 1112137)*

*“I’ve heard other people say anemia is when there’s high blood pressure and dizziness.”*

*(Mother, resettlement community, 1112123)*

In other cases, caregivers pointed to behavioral changes as indicators of an anemic child.

*“When nutrition is bad, when (the child) doesn’t eat well, when nutrition is uneven, that sort of thing (can cause anemia).”*

*(Mother, resettlement community, 1112115)*

*“I think if the baby is anemic, the baby will cry a lot, misbehave, be uncomfortable, be noisy. The baby’s mood will be unstable. The baby’s constitution will be bad and droopy. They’ll be dispirited.”*

*(Grandmother, migrant community, 1221116)*

The failure of caregivers to assess whether their child is anemic may be one reason that some caregivers were not addressing the condition. In fact, exactly one-third of caregivers (28/84) plainly stated that the child in their care was not anemic—while according to our data, the child was suffering from some level of anemia.

*“My child is in good health.”*

*(Mother, resettlement community, 1112110. Child moderately anemic, Hb = 74)*

*“My child is … fine.”*

*(Mother, migrant community, 1221145. Child moderately anemic, Hb = 99)*

*“No way. My granddaughter is not anemic.”*

*(Grandmother, western China rural village, 1402116. Child moderately anemic, Hb = 82)*

**Sub-Hypothesis 2d.** *The misinformation is due to the fact that caregivers do not understand how to treat anemia.*

Given that the general understanding among caregivers is that anemia is related to nutrition, many of the proposed treatments also had to do with feeding behavior (however, few identified the correct interventions):

*“Doesn’t it have to do with (the child) being picky? And not giving the child well-arranged servings of rice, meat, and vegetables, giving (those things) unevenly. But exactly what causes anemia, I’m not too sure about that.”*

*(Grandmother, migrant community, 1221119)*

*“I would provide food that is good for the child’s stomach. But I’m really not clear what food that is exactly. If the child is only a little anemic, I would not try to manage it.*

*(Mother, migrant community, 1221329)*

*“You should give the child lots of water to drink.”*

*(Grandmother, migrant community, 1221119)*

Others believe that there is no need to treat anemia:

*“My child had a full body exam at the hospital when she was born, so she’s fine. She does not need treatment.”*

*(Mother, migrant community, 1221145. Child moderately anemic, Hb = 99)*

*“No, (she is not anemic). But, even if she were anemic, nothing is needed be done.”*

*(Grandmother, western China rural village, 1402116. Child is moderately anemic, Hb = 82)*

The limited understanding of anemia by caregivers may be due to the fact that they have minimal access to sources of information to educate them on this condition. However, the caregivers actually told the interviewers that their information came from many different sources. Fifteen caregivers said they learned about anemia on the internet (often via cell phones), 29 cited hospitals and doctors, 22 people said they mainly listened to what others—typically family members—told them, and 21 said they had learned about anemia almost solely through their personal experience. Caregivers in our subpopulations rely on all of the above sources.

*“I use the internet on my phone to learn about (anemia.) But there are many different opinions so I do not know which one is right.”*

*(Mother, western China rural village, 1402120)*

*“I listen to the village doctor. He said that if the child’s face is yellow, he has anemia and that this is bad for the child.”*

*(Mother, western China rural village, 1112110)*

*“I listen to my family and close friends in the village. They say that it is easy to know if your child has anemia. You just look at your child and see if they’re dizzy.”*

*(Mother, western China rural village, 1406103)*

*“I don’t trust anyone else. It’s just based on my own knowledge.”*

*(Mother, western China rural village, 1402101)*

*“The information comes from my own life, and from my cell phone.”*

*(Father, migrant community, 1221328)*

**Hypothesis 3.** *Anemia is a problem because of constraints such as poverty or the absence of a functioning health care system, and not because caregivers do not understand what it is or how to address it.*

Even if individuals understood what anemia was and how to identify and treat it, it could be that there are other factors preventing caregivers from treating their anemic child. While there may be many potential constraints, we address two in this section: the poor environment in which many families live, and the quality of the rural health system.

#### 3.2.1. The Environment in Poor, Rural China

While the issue in rural China for most families is not deep poverty, it is possible that the living environment—the nature of local infrastructures and access to markets for the foods necessary for a nutritious diet—is still an issue. In fact, this was mostly found to be true in western rural communities and resettlement communities. Since insufficient intake of red meat is the most common cause of iron-deficiency anemia for infants/toddlers in developing countries [40,41,42], our interview questions focused on access to meat, rather than other foods that might be rich in iron. Of the eight respondents who reported having difficulty accessing nutritious foods (e.g., iron-rich red meats and fish), all of them were from these subpopulations.

*“It is inconvenient to buy meat. After all, there is still some distance from the county seat to my home.”*

*(Grandmother, western China rural village, 1402114)*

*“It isn’t convenient to buy meat here. The store that sells meat is too far away.”*

*(Mother, resettlement community, 1112116)*

*“It’s inconvenient. You have to go to the main road to buy (meat), it’s so far.”*

*(Mother, resettlement community, 1112114)*

#### 3.2.2. Absence of Quality Health Care

When asked how they would judge whether their child had anemia, the majority of caregivers (50–60%) said they would only know if they had an examination done at the hospital. Migrant community caregivers were most likely to give this response (83% of migrant caregivers responded this way); central rural village caregivers were least likely to give this response (43% said they needed a hospital exam to judge whether their child had anemia). These responses indicate that at least some interviewees rely on the health system to provide care.

However, the quality of health care in rural areas is poor. When using a standardized patient approach, only 26% percent of doctors were able to correctly diagnose the condition of a child [40]. Even fewer doctors treated the patient properly. In fact, in some communities, the probability of becoming even more sick after visiting a doctor was greater than the probability of receiving adequate treatment [43].

Indeed, a number of caregivers said they would not bring their child to the hospital because they were worried it might do more harm than good:

*“Sometimes when you see the doctor, the anemia medicine they prescribe would make (the child) feverish …”*

*(Grandmother, central China rural village, 1231124)*

*“The hospital isn’t very good. If we went to the hospital (my child) might get sick in another way.”*

*(Mother, western China rural village, 1402118)*

*“I would never bring a child to the hospital …”*

*(Grandmother, migrant community, 1221126)*

## 4. Discussion

Using samples from five provinces, this paper maps out anemia rates among infants and toddlers in four major rural subpopulations in China. The overall prevalence of anemia among the full weighted sample of infants/toddlers is 43%. To the extent that our samples are representative of the four rural subpopulations that are being studied, this means that slightly under half of all young rural children (ages 6 months to 30 months) living in western China rural communities, resettlement communities, central China rural communities, and migrant communities in large cities suffer from micronutrient deficiencies. Since rural infants and toddlers in these subpopulations make up over 70% of all rural children in the 6-month to 30-month age cohorts, even if rural infants/toddlers in the rest of rural China’s subpopulations (those in eastern China’s rural villages and those that live in the suburbs of large cities—groups that we did not study in this paper) were not anemic at all, this means more than three out of ten rural young children—who account for around 75% of all of China’s infants/toddlers—are anemic.

The anemia rates we report are high compared to those found in developed, high-income countries. According to the World Health Statistics, the prevalence of anemia in countries such as Canada (9%), United States (9%), and Germany (12%) are much lower than those found in the four rural subpopulations studied in this paper [44]. This is consistent with findings cited in our introduction; namely, that Chinese children were anemic at rates tens of percentage points higher than children from high-income countries worldwide [16,20].

While the anemia prevalence found in our results is high by the standards of developed countries, it perhaps should not be surprising when comparing rural China to other developing countries. In fact, anemia rates among young children in poor countries across the world are often higher than those observed in our sample. For example, in Burkina Faso, a low-income Sub-Saharan African country, rates of anemia are as high as 91.6% among children aged 6 to 59 months [45]. Interestingly, the rates that we report in this paper are not too different than those that are found in several middle-income countries (that is, countries with GDP/capita levels similar to those of China). Specifically, reports from the Central Intelligence Agency find that the anemia rates for children from subset of middle income countries (Botswana: 40%; Maldives: 39%; South Africa: 37%) are similar to the rates we find for rural Chinese infants and toddlers [46]. Our finding is also consistent with the findings of Li et al. [47], that China’s levels of micronutrient deficiencies among different age cohorts than those being studied here (e.g., ages 3 to 6 years and among adult women) are also similar among middle-income countries.

An important finding of our paper that has not been reported previously in the literature is that anemia rates are high throughout all of the four rural subpopulations being studied. The measured rates of anemia in the four different communities are 40%, 52%, 46%, and 43% for the western rural communities, resettlement migration communities, central rural communities, and migrant communities, respectively. While previous recent works done on anemia [15,16,17,18] were from communities in western China’s rural villages, the findings in this paper show that these are not areas with extraordinarily high rates of anemia. In fact, the point estimate of anemia of infants/toddlers in western rural communities is lower than those in all of the other communities studied. This finding is particularly surprising in light of literature that indicates that anemia rates are negatively associated with income [48], since income per capita of families is highest in migrant communities and lowest in western China rural villages [47].

The absence of any significant correlations between demographic characteristic and anemia rates across macroregions is supported by correlation analysis across respondents and their families within subpopulation samples. This finding could be consistent with an interpretation of our results that the micronutrient problem in these rural communities is fundamentally due to an absence of information that affects all subpopulations of rural society (and not some other systematic barrier). According to our analysis, when examining the factors that might be expected to be correlated with anemia, we find that there is not any close association between anemia and the maternal education level; there also is no systematic relationship either between a household’s asset holdings or whether the family receives social security (that is, whether they are poor) and the prevalence of anemia. In fact, the only variable in our analysis that is systematically associated with anemia is the age of the infants and toddlers. Across the entire sample, the prevalence of anemia decreased from 51% (for infants aged 6 to 18 months) to 30% (for infants aged 18 to 30 months) in our data. This is consistent with other previous studies [49]. This previous work (which studied western China rural village population) found that as a cohort of infants/toddlers (that were not part of any intervention) ages and their diets transitioned, hemoglobin concentrations gradually rose and the prevalence of anemia fell. Specifically, consumption of all food categories increases as infants/toddlers get older. Older toddlers are more frequently fed vegetables or fruits rich in vitamin A than younger children. Consumption of iron-rich foods including meat and fish, beans, nuts, and seeds also increases gradually with age [49]. Additionally, this work suggests that misinformation about anemia and healthy feeding practices for young children could be at least partly behind the high rates of micronutrient deficiencies in rural China.

## 5. Conclusions

Our data indicates that the fundamental nutrition problem in rural China today is one of micronutrient deficiencies and not poverty-induced malnutrition, which would include macronutrient deficiencies. We find low rates of stunting (4% overall), underweightness (1% overall), and wasting (4% overall). In no subpopulations did the rates for any of these indicators exceed 5%. The contrast in our data between high anemia prevalence and low rates of stunting, underweightness, and wasting suggest that the infants in rural China suffer from an absence of a diverse, micronutrient-rich diet rather than a simple problem of insufficient calories. Other researchers have drawn similar conclusions in individual subpopulations [15,16], although, again, this paper shows such findings are common in at least four of rural China’s subpopulations.

So what evidence does this study present that can help identify why it is that so many infants and toddlers are anemic? Our qualitative interviews provide evidence that a poor understanding of anemia—along many different dimensions—could be the fundamental source of the problem. Even though most caregivers (78 of the 84 qualitative interviewees) have heard of anemia, many do not understand its causes. They lack an understanding of the effects of anemia on infants and toddlers. They do not know what the symptoms of anemia are (or are not). Furthermore, most caregivers do not understand how the problem of anemia in a child should be addressed.

Our study did find that in some rural communities, there are other, more exogenous constraints than misinformation. For example, some caregivers cited high transaction costs in obtaining fresh meat and poor quality of rural health care as obstacles to caring for their children. However, because income levels and access to transport services are not extremely low (as there were in China several decades ago and as they are in many poor countries today), these are not fundamental binding constraints. Furthermore, these problems were cited infrequently in comparison to the number of times caregivers answered our questions with misinformation about anemia.

The major contribution of this paper, which presents results from a large sample using mixed-methods analysis, is to demonstrate that anemia is found at very high rates among rural Chinese infants and toddlers living in a variety of geographic conditions. Although anemia rates in rural China are not as alarmingly high as they are in very low-income developing countries, China still lags far behind developed countries in this dimension of nutrition and health. Because anemia can have negative impacts on the development outcomes of large shares of the nation’s future population, the government should focus resources and effort on reducing infant anemia rates in rural China. Our qualitative results indicate that interventions should include massive information campaigns to improve how rural caregivers understand, respond to, and prevent anemia in their children. If such interventions were accompanied in some of China’s poorest regions by direct nutrition interventions (or assistance in having current programs work more effectively), the efforts to overcome anemia and its related problems would likely be more successful in those areas where rural infrastructure and health care are still weak.

This paper does have limitations. While this represents the first study in recent years to systematically sample caregivers and their infants/toddlers across four rural subpopulations, and while the samples were all randomly chosen, the sample sizes are still small and coverage is limited. This is especially true for the three subsamples outside of western China’s rural villages. Given the results reported in this paper, a more comprehensive study with a much larger and systematic sampling plan would be valuable to confirm the overall prevalence of anemia and its sources in rural China. 

## Figures and Tables

**Table 1:** Summary of Project 1 to Project 5.

Study	Location of Study	Date	Community Type	Ages of Children	Number of Observations
Project 1	Southern Shaanxi	2015–2016	Western Rural Communities	6–24 months	1804
Project 2	Hebei; Yunnan	2015–2016	Western Rural Communities	6–30 months	638
Project 3	Southern Shaanxi, Henan	2017	Resettlement Migration Communities	6–30 months	135
Project 4	Henan; Central Shaanxi	2017	Central China Rural Communities	6–30 months	128
Project 5	Beijing; Zhengzhou, Henan; Xi’an, Shaanxi	2017	Migrant Communities	6–30 months	204

Data source: Authors’ survey. Notes: In this paper we make use of five data sets that have been collected by the authors. In total there were five data collection efforts that focused on collecting data in one of four different rural sub-populations—western rural communities; resettlement communities; central China rural communities; and migrant communities.

**Table 2:** Summary statistics.

Variables	Full Sample	Western Rural Villages	Resettlement Migration Communities	Difference (2)−(3)	Central China Rural Villages	Difference (2)−(5)	Migrant Communities	Difference (2)−(7)
	Mean (SD) (1)	Mean (SD) (2)	Mean (SD) (3)	*p*-Value (4)	Mean (SD) (5)	*p*-Value (6)	Mean (SD) (7)	*p*-Value (8)
*Characteristics of the infants*								
Age (in month)	16.50	15.44	16.41	0.330	17.62	0.001	16.09	0.524
	(6.73)	(5.72)	(6.80)		(7.39)		(6.74)	
Gender (1 = male)	0.53	0.51	0.54	0.885	0.56	0.659	0.49	0.961
	(0.50)	(0.50)	(0.50)		(0.50)		(0.50)	
Premature (1 = yes)	0.06	0.05	0.04	0.983	0.06	0.849	0.09	0.025
	(0.24)	(0.21)	(0.19)		(0.24)		(0.29)	
*Characteristics of the parents and the households*								
Maternal age	0.70	0.67	0.71	0.802	0.70	0.949	0.75	0.137
(1 = above 25 years old)	(0.46)	(0.47)	(0.45)		(0.46)		(0.43)	
Maternal education level	0.34	0.23	0.36	0.009	0.32	0.143	0.61	<0.001
(1 = 12 years or higher)	(0.47)	(0.42)	(0.48)		(0.47)		(0.49)	
Primary caregiver	0.67	0.73	0.58	0.004	0.59	0.012	0.73	0.999
(1 = mother)	(0.47)	(0.45)	(0.50)		(0.49)		(0.44)	
Household receives social	0.11	0.11	0.11	0.996	0.13	0.971	0.09	0.791
security support (1 = yes)	(0.32)	(0.32)	(0.31)		(0.33)		(0.28)	
Household asset index	0.36 (1.21)	−0.19 (1.21)	0.37 (1.22)	<0.001	0.48 (1.08)	<0.001	1.23 (0.86)	<0.001
Observations	2909	2442	135		128		204	

Data source: Authors’ survey. Notes: Household asset index is constructed using polychoric principal components on the following variables: tap water, toilet, water heater, washing machine, computer, internet, refrigerator, air conditioner, motor or electronic bicycle, and car. We calculate overall summary statistics using sampling weights for each observation. The proportions for each subpopulation in rural China are 37.7% for western China rural communities, 1.4% for resettlement migration villages, 42.0% for central China rural communities, and 18.8% for migrant communities. We calculate the sampling weights using the following formula: sampling weight = proportion of subpopulation in total population/proportion of subpopulation in sample. In our data, the subpopulation proportions in the sample are the following: 84.0% for western China rural communities, 4.6% for resettlement migration villages, 4.4% for central China rural communities, and 7.0% for migrant communities. Therefore, the sampling weight for western China rural communities is 0.44 (which is equivalent to 37.7%/84%), the sampling weight for resettlement migration villages is 0.3 (which is equivalent to 1.4%/4.6%), the sampling weight for central China rural communities is 9.55 (which is equivalent to 42%/4.4%), and the sampling weight for migrant communities is 2.69 (which is equivalent to 18.8%/7%). We created a couple of dummy variables to capture the characteristics of children, their parents and households. We let the variable “gender” equal 1 if the child is male; variable “premature” equal 1 if the child was a premature; variable “maternal age” equal 1 if the mother’s age is older than 25; variable “maternal education level” equal 1 if the mother has attained over 12 years education; variable “primary caregiver” equal 1 if the child’s primary caregiver is the mother; variable “household receives social security support” equal 1 if the household receives social security support. 0 is otherwise. In columns 4, 6, and 8, we report the *p*-values of the differences in characteristics between communities, taking the western rural villages as the control. We do not report the absolute differences in these characteristics.

**Table 3:** Health outcomes of infants.

Variables	Full Sample	Western Rural Villages	Resettlement Migration Communities	Difference (2)−(3)	Central China Rural Villages	Difference (2)−(5)	Migrant Communities	Difference (2)−(7)
	Mean (SD) (1)	Mean (SD) (2)	Mean (SD) (3)	*p*-Value (4)	Mean (SD) (5)	*p*-Value (6)	Mean (SD) (7)	*p*-Value (8)
Anemic (Hb < 110 g/L)	0.43	0.40	0.52	0.118	0.46	0.716	0.43	0.947
	(0.50)	(0.49)	(0.50)		(0.50)		(0.50)	
Stunting (Height for age *z*-score < −2)	0.04	0.05	0.05	1.000	0.03	0.921	0.02	0.490
	(0.19)	(0.21)	(0.22)		(0.18)		(0.15)	
Underweight (Weight for age *z*-score < −2)	0.01	0.02	0.02	0.999	0.02	0.999	0.00	0.352
	(0.12)	(0.13)	(0.12)		(0.13)		(0.00)	
Wasting (Weight for height *z*-score < −2)	0.04	0.03	0.02	0.991	0.04	0.914	0.04	0.914
	(0.18)	(0.17)	(0.15)		(0.20)		(0.19)	
Observations	2842	2380	127		125		184	

Data source: Authors’ survey. Notes: We calculate the statistics using sampling weights for each observation. The proportions for each subpopulation in rural China are 37.7% for western China rural communities, 1.4% for resettlement migration villages, 42.0% for central China rural communities, and 18.8% for migrant communities. We calculate the sampling weights using the following formula: sampling weight = proportion of subpopulation in total population/proportion of subpopulation in sample. In our data, the subpopulation proportions in the sample are the following: 84.0% for western China rural communities, 4.6% for resettlement migration villages, 4.4% for central China rural communities, and 7.0% for migrant communities. Therefore, the sampling weight for western China rural communities is 0.44 (which is equivalent to 37.7%/84%), the sampling weight for resettlement migration villages is 0.3 (which is equivalent to 1.4%/4.6%), the sampling weight for central China rural communities is 9.55 (which is equivalent to 42%/4.4%), and the sampling weight for migrant communities is 2.69 (which is equivalent to 18.8%/7%).

**Table 4:** Health outcomes of infants at different age ranges.

Health Outcomes	Age (6–18 Months)	Age (18–30 Months)	Difference (1)–(2)
	Mean (SD) (1)	Mean (SD) (2)	*p*-Value (3)
Anemic (Hb < 110 g/L)	0.51	0.30	<0.001
	(0.50)	(0.46)	
Stunting (Height for age *z*-score < −2)	0.04	0.03	0.199
	(0.20)	(0.17)	
Underweight (Weight for age *z*-score < −2)	0.01	0.01	0.959
	(0.12)	(0.12)	
Wasting (Weight for height *z*-score < −2)	0.04	0.03	0.181
	(0.19)	(0.17)	
Observations	1878	967	

Data source: Authors’ survey. Notes: We calculate the statistics using sampling weights for each observation. The proportions for each subpopulation in rural China are 37.7% for western China rural communities, 1.4% for resettlement migration villages, 42.0% for central China rural communities, and 18.8% for migrant communities. We calculate the sampling weights using the following formula: sampling weight = proportion of subpopulation in total population/proportion of subpopulation in sample. In our data, the subpopulation proportions in the sample are the following: 84.0% for western China rural communities, 4.6% for resettlement migration villages, 4.4% for central China rural communities, and 7.0% for migrant communities. Therefore, the sampling weight for western China rural communities is 0.44 (which is equivalent to 37.7%/84%), the sampling weight for resettlement migration villages is 0.3 (which is equivalent to 1.4%/4.6%), the sampling weight for central China rural communities is 9.55 (which is equivalent to 42%/4.4%), and the sampling weight for migrant communities is 2.69 (which is equivalent to 18.8%/7%).

**Table 5:** Correlations between anemia and infant/household characteristics.

Variables	Anemic
Child characteristics	
Child age (months)	−0.01 *** (0.00)
Male (1 = yes)	−0.04 (0.03)
Premature (1 = yes)	0.00 (0.07)
Household characteristics	
Maternal age (1 = above 25 years old)	−0.04 (0.05)
Maternal education level (1 = 12 years or higher)	−0.05 (0.03)
Primary caregiver (1 = mother)	0.07 (0.05)
Household receives social security support (1 = yes)	−0.03 (0.08)
Household asset index	−0.01 (0.02)
Western rural community (1 = yes)	−0.07 (0.21)
Resettlement migration community (1 = yes)	0.11 (0.09)
Central rural community (1 = yes)	0.10 (0.07)
Tester Fixed Effect	YES
Observations	2682
Adj. R^2^	0.139

Data source: Authors’ survey. *** *p* < 0.01. Notes: We created a couple of dummy variables to analyze the correlationship. We let the variable “gender” equal 1 if the child is male; variable “premature” equal 1 if the child was a premature; variable “maternal age” equal 1 if the mother’s age is older than 25; variable “maternal education level” equal 1 if the mother has attained over 12 years education; variable “primary caregiver” equal 1 if the child’s primary caregiver is the mother; variable “household receives social security support” equal 1 if the household receives social security support; variable “western rural community” equal 1 if the observation is from western rural community; variable “resettlement migration community” equal 1 if the observation is from resettlement migration community; variable “central rural community” equal 1 if the observation is from central rural community. 0 is otherwise. Household asset index is constructed using polychoric principal components on the following variables: tap water, toilet, water heater, washing machine, computer, internet, refrigerator, air conditioner, motor or electronic bicycle, and car. We calculate overall summary statistics using sampling weights for each observation. The proportions for each subpopulation in rural China are 37.7% for western China rural communities, 1.4% for resettlement migration villages, 42.0% for central China rural communities, and 18.8% for migrant communities. We calculate the sampling weights using the following formula: sampling weight = proportion of subpopulation in total population/proportion of subpopulation in sample. In our data, the subpopulation proportions in the sample are the following: 84.0% for western China rural communities, 4.6% for resettlement migration villages, 4.4% for central China rural communities, and 7.0% for migrant communities. Therefore, the sampling weight for western China rural communities is 0.44 (which is equivalent to 37.7%/84%), the sampling weight for resettlement migration villages is 0.3 (which is equivalent to 1.4%/4.6%), the sampling weight for central China rural communities is 9.55 (which is equivalent to 42%/4.4%), and the sampling weight for migrant communities is 2.69 (which is equivalent to 18.8%/7%). We also control for Bayley tester fixed effects. All standard errors account for clustering at the village level.
